# Analysis of some determining factors of consanguinity in North Morocco: a cross-sectional epidemiological study

**DOI:** 10.11604/pamj.2024.49.133.34881

**Published:** 2024-12-23

**Authors:** Houria Hardouz, Mustapha Zghaid, Amine Arfaoui, Ali Quyou

**Affiliations:** 1 Faculty of Sciences, Ibn Tofaïl University, Kenitra, Morocco,; 2 Royal Institute of Managers Training, Sale, Morocco,; 3 Institute of Sport Professions, Ibn Tofail University, Kenitra, Morocco

**Keywords:** Consanguinity, isonymy, determining factors, North Morocco

## Abstract

**Introduction:**

consanguinity represents a serious concern for public health. It represents 20 to 55% of unions in the Middle East, North Africa, and Central Asia. The present work aims to find out some determining factors of consanguinity and isonymy in the population of North Morocco.

**Methods:**

it consists of a cross-sectional epidemiological study conducted on 238 couples from the region of Tanger-Tetouan located northwest of Morocco. The studied variables were consanguinity, isonymy, union type, geographical origin, education level, profession, ethnicity, and belonging to Shorfas.

**Results:**

the results showed that consanguineous couples represent 45.4%. Among them, unions between first cousins are predominant with 91%, among which the union with the daughter of the father´s brother represents 56%. The risk analysis showed that both husbands and wives with higher education levels display significantly lower risk of being in a consanguineous union (OR=0,36; IC 95% = 0,19- 0,65). Furthermore, wives of urban origin have a significantly lower chance of being in a consanguineous union (OR=0, 51; IC 95% =0,26-1,011). Regarding isonymy, we found that belonging to Shorfas is significantly associated with isonymous unions in both husbands and wives (p=0.04 and p=0.001 respectively). Moreover, urban-origin males display a significantly lower chance of being in isonymous unions (OR=0.35; IC 95% =0.15- 0.80).

**Conclusion:**

health authorities should make more efforts to raise the awareness of young people of the health disorders caused by consanguineous marriages.

## Introduction

Consanguinity is a common practice in many regions of the world. It represents 20 to 55% of unions in the Middle East, North Africa, and Central Asia [1-3]. Several studies have reported high consanguinity rates in Morocco [4,5]. This practice remains frequent in spite of modernization and literacy [6], which is probably due to many factors, such as safeguarding familial properties, facilities for wedding arrangements, good relationships with parents-in-law, and financial benefits related to dowry [7-9]. Consanguinity represents a serious concern for public health. Indeed, it has been demonstrated that descendants of consanguineous unions have a higher risk of genetic disorders because of the expression of autosomal recessive mutations inherited from a common ancestor [10,11]. The aim of the present work is to bring out the rates of consanguinity and isonymy, and to find out if geographical origin, education level, profession, and ethnicity are determining factors for this practice in the population of North Morocco.

## Methods

**Study design:** the present work consists of a cross-sectional epidemiological study conducted in the region of Tanger-Tetouan located northwest of Morocco.

**Study setting:** the region of Tanger-Tetouan-Al Hoceima is located in the extreme northwest of Morocco over an area of 17,262 km^2^. It is limited to the north by the Strait of Gibraltar and the Mediterranean, to the west by the Atlantic Ocean, to the south-west by the Rabat-Salé-Kenitra region, to the south-east by the Fes-Meknes region, and to the east by the Oriental region. In 2014, the regional urban environment was home to 2,131,725 inhabitants, compared to 1,425,004 in rural areas. Thus, 93.9% of the increase in the regional population during the period 2004-2014 is due to the increase in the urban population. Indeed, the annual urban population growth rate is 2.45% against only 0.21% in rural areas [12].

**Study participants:** married couples, of all ages and all socioeconomic categories, living in Tanger-Tetouan region.

### Variables

**Dependent variable**

**Consanguinity:** a consanguineous union is a union in which the husband and the wife are relatives. The consanguinity was not determined through the pedigree construction but through self-reported relationships between spouses.

**Independent variables:** isonymy, which refers to unions between spouses with the same surname; natural family link between spouses; geographical origin; education level; profession; ethnicity; belonging or not to “Shorfa”. Ethnicity is defined as a group of persons who identify with each other according to common attributes that distinguish them from other groups. These attributes include common traditions, ancestry, language, history, and religion [13]. The word “Shorfa” refers to a group of people with common values of honor, nobility, and notoriety. They claim to be the descendants of the prophet Muhammad and his daughter Fatima Zahrae [14].

**Data collection and sample size:** we used convenience sampling for collecting data. This is a non-probabilistic technique frequently used in quantitative studies. Inconvenience samples, the fact that the opportunity to participate is not equal for all individuals in the target population makes the study results not necessarily generalizable to this population. The research tool was a randomly administered questionnaire containing questions about: personal and sociodemographic information of both husbands and wives, the relationship between them, pregnancy loss, and health problems in children. The study lasted for three months, from January to March 2015. The questionnaires were randomly administered to 385 couples in public places, and respondents, who were either husbands, wives or both, all expressed their will to participate in the study through written consent. After the examination of completed questionnaires, we eliminated 147 ones in which the dependent variable (consanguinity) was not provided. Thus, only 238 questionnaires were analyzed.

**Inclusion criteria:** couples living in Tanger-Tetouan-Al Hoceima is a region and at the survey period; ii) couples married for over one year.

**Exclusion criteria:** undefined or unclearly defined relationship between spouses, ii) couples not living in Tanger-Tetouan-Al Hoceima region at the survey period.

**Data analysis:** the statistical analysis, which was performed using SPSS 25.0., was organized in two parts. In the first part, we described the characteristics of the studied couples according to each of the independent variables. In the second part, an analytical study was conducted to evaluate the relationship between consanguinity, isonymy and each of the independent variables. For this purpose, the Chi-squared test was used to determine the association significance, Cramer V test to measure the effect size, and the Odds Ratio test (OR) to evaluate the risk of being in consanguineous union/isonymous union according to each independent variable. The Chi-squared test is considered significant when the p-value is lower than 0.05, whereas the OR test is considered as significant when the confidence interval (CI) does not include the value 1. The effect size is considered as weak when Cramer V is lower than 0.2, moderate when it is between 0.2 and 0.6, and high when it is greater than 0.6.

The calculation of the consanguinity coefficient was performed in two ways: i) Genealogy-based consanguinity coefficient: the consanguinity coefficient of an individual i (Fi) is the probability that the two homologous genes he has at a given locus be identical by Mendelian progeny (inherited from the same common ancestor). It refers to the degree of kinship of the two parents, the father P and the mother M of the individual i. If parents are not relatives then Fi=0 (Table 1). Fi is estimated as follows:


$$Fi=\sum \left(1/2\right)n+m+1\left[\left(1+FA\right)\right]$$

n and m are respectively the number of generations that separate Pand M from the ancestor A; FA is the consanguinity coefficient of the common ancestor A [15]. ii) Mean consanguinity coefficient in the population: it is the average of consanguinity coefficients of all individuals of a population. It is calculated by Bernstein coefficient:


Ca=∑fiFI

fi: relative frequency of couples having a kinship measured by FI which is their child´s consanguinity coefficient [16]. The probability of isonymy is equal to the value consanguinity coefficient multiplied by 4 [17].

**F=P/4**

P: number of isonymous unions/number of total unions; F: consanguinity coefficient multiplied.

**Table 1:** consanguinity coefficients according to union types

Consanguineous Kinship types	First Cousins	Second cousins	First cousin once removed	Double first cousins	Double second cousins	uncle-niece
Consanguinity coefficients	F=1/16 =0,0625	F =1/64 =0,0156	F=1/32 =0,0313	F=1/8 =0,125	F=1/32 =0,0313	F=1/8 =0,125

## Results

### Descriptive analysis

**Socio-demographic and cultural characteristics:** results showed that most studied couples are of urban origin with 85.5% in males and 81.5% in females. Regarding education level, illiteracy is much higher in wives than in husbands with 40% and 9% respectively. It should also be noted that only 15% of studied wives reached university level compared with 30% of husbands (Table 2). According to the profession, 45% of male spouses have intermediate to high-level positions whereas 75% of female spouses are jobless (Table 2). With regard to ethnicity, more than 77% of spouses are Arab whereas 21% are Berbers. Furthermore, almost 11% of studied couples belong to the category of Shorfa (Table 2).

**Table 2:** socio-demographic and cultural characteristics of studied couples

	Husbands		Wives	
	Number	%	Number	%
**Geographical origin**				
Urban	194	85.5	189	81.5
Rural	33	14.5	43	18.5
Total	227	100.0	232	100.0
Missing data	11	4.6	6	2.5
**Education level**				
Illiterate	21	8.9	88	39.6
Primary	72	30.5	30	13.5
Secondary	72	30.5	71	32.0
University	71	30.1	33	14.9
Total	236	100.0	222	100.0
Missing data	2	0.8	16	6.7
**Profession**				
Without	2	0.9	174	76.3
Entrepreneurs, tradesmen and craftsmens	43	18.3	2	0.9
Farmers	7	3.0	-	-
Managerial and intellectual professions	56	23.8	21	9.2
Intermediate professions	52	22.1	26	11.4
Employees	29	12.3	-	-
workers	46	19.6	5	2.2
Total	235	100.0	228	100.0
Missing data	3	1.3	10	4.2
**Ethnicity**				
Arab	182	77.8	187	79.6
Berber	52	22.2	48	20.4
Total	234	100.0	235	100.0
Missing data	4	1.7	3	1.3
**Shorfa**				
Not Shorfa	186	88.6	187	89.9
Shorfa	24	11.4	21	10.1
Total	210	100.0	208	100.0
Missing data	28	11.8	30	12.6

**Figure 1:**
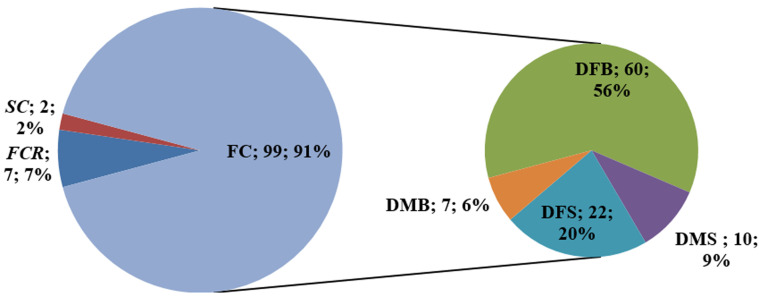
repartition according to the family relationship between spouses; FC: first cousin; FCR: first cousin removed; SC: second cousin; DFB: daughter of father's brother; DFS: daughter of father’s sister; DMS: daughter of mother's sister; DMB: daughter of mother's brother

**Repartition according to the consanguinity and isonymy:** results showed that consanguineous couples represent 45.4% of all studied couples. Among them, unions between first cousins (FC) are predominant at 91%, among which the union with the daughter of the father´s brother (DFB) is most frequent at 56% (Figure 1). Regarding isonymy, the study showed that 21% of couples are isonymous with 49 cases.

### Analysis of consanguinity determining factors

**Geographical origin:** the analysis of the relationship between consanguinity and geographical origin in wives demonstrated a statistically significant and weak association (p=0.05; V=0.12). Indeed, consanguinity is more frequent (58%) in wives of rural origin. That was confirmed by the significant Odds ratio between origin and consanguinity (OR= 0, 51; IC 95% = 0,26-1,011) which implies that wives of urban origin have a significantly lower chance of being in consanguineous union (Table 3). On the other hand, no significant relationship was found between geographical origin and consanguinity in husbands.

**Table 3:** analysis of association between consanguinity and studied determining factors

	Husbands			Wives		
Factors	Khi2	P-value	V de Cramer	Khi2	P-value	V de Cramer
Geographical origin	3.06	0.08	0.11	3.78	0.05*	0.12
Education level	13.87	0.003*	0.24	1.06	0.007*	0.23
Profession	3.61	0.09	0.21	4.49	0.28	0.14
Ethnicity	0.54	0.46	0.04	0.74	0.38	0.05
Shorfas	0.42	0.51	0.04	3.12	0.07	0.12

**Education level:** in husbands, the analysis of the relation between consanguinity and education level displayed a statistically significant association (p=0.003) with moderate intensity (V=0.24). Indeed, consanguineous unions are less frequent in men with university education levels, with 18.9% compared with 81.1% in men who are illiterate or with low education levels. The risk analysis showed that husbands with higher education levels present a significantly lower risk of being in a consanguineous union (OR=0,36; IC 95% = 0,19- 0,65) (Table 3). In wives, the association between consanguinity and education level was also significant (p=0.007), with moderate intensity (V= 0.22). In fact, only 5.37% of women with higher education levels are in consanguineous unions, compared with 94.63% of women with low education levels. Through the risk analysis we found that wives with higher education levels present a significantly lower risk of being in a consanguineous union (OR=0.20; IC 95% = 0.075- 0.55) (Table 3).

**Other factors:** the statistical analysis showed no association between consanguinity and the other studied factors which are profession, ethnicity, and belonging to Shorfas (Table 3).

**Analysis of isonymy determining factors**

**Geographical origin:** in male spouses, the relationship between isonymy and geographical origin was shown to be statistically significant but weak (p=0.01; V=0.17). Indeed, husbands of rural origin were more frequent in isonymous unions compared with non-isonymous unions, with 27% and 11% respectively. Risk analysis showed that urban-origin males display a significantly lower chance of being in isonymous union (OR=0.35; IC 95% =0.15- 0.80) (Table 4). As far as female spouses are concerned, no significant association was found between isonymy and geographical origin (p=0.27).

**Table 4:** analysis of association between isonymy and other determining factors

	Husbands			Wives		
Factors	Khi2	P-value	V de Cramer	Khi2	P-value	V de Cramer
Geographical origin	6.53	0.01*	0.17	1.22	0.26	0.07
Education level	0.19	0.97	0.02	3.13	0.37	0.11
Profession	3.43	0.76	0.12	15.91	0.006	0.09
Ethnicity	0.89	0.34	0.062	0.84	0.35	0.06

**Belonging to Shorfas:** the relationship between isonymy and belonging to Shorfas was statistically significant for both male and female spouses (p=0.04 and 0.001 respectively). The intensity of association was weak for the first and moderate for the second (V=0.14 and 0.26 respectively). Indeed, Shorfas belonging spouses were more represented in isonymous unions than in non-isonymous ones with respectively 23% and 9% in males, and 30% and 7% in females (Table 4). The risk analysis showed that Shorfas spouses, both husbands and wives, present a significantly higher risk of being in isonymous union. However, that risk was higher in wives with an OR of 5.9 [2.13-16.13] compared with 2.9 [1.03- 8.24] in husbands (Table 4).

**Figure 2:**
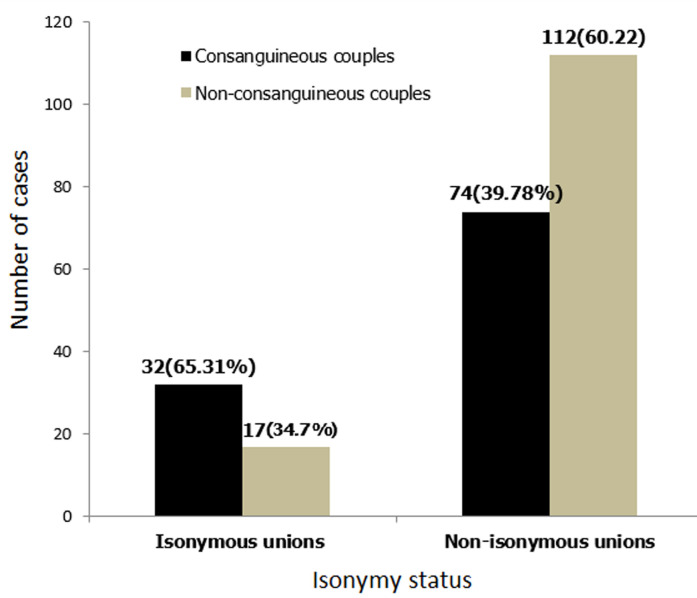
repartition of couples according to consanguinity and isonymy

**Analysis of the interaction between consanguinity and isonymy:** the present study showed that among isonymous unions, 65.31% of couples are consanguineous, whereas only 39.78% of couples are consanguineous among non- isonymous unions. The association between isonymy and consanguinity was statistically significant (p=0.001) (Figure 2).

**Inbreeding coefficient:** according to the data, the inbreeding coefficient calculated using genealogy is 0.0173. This coefficient is equal to 0.0521 if it is calculated using isonymy.

## Discussion

The present work aimed to describe consanguinity and find out some of its determining factors in the north of Morocco. According to the obtained results, consanguineous unions are still a common practice in this region of the country, as in many other countries, especially in Africa, the Middle East, and West Asia [18]. Indeed, about 1.1 billion people in the world live in consanguineous unions [10], and this type of marriage is particularly common in Maghreb countries despite modernization [19]. The consanguinity rate found in the present study is higher than that registered in other regions in Morocco and elsewhere. It is about 20% in Brazil [20], 15% in Chili [21], 6% in Spain [22], 39% in Tiflet-Morocco [5], and 36% in Azgour-Morocco [23]. Nevertheless, it remains lower than in some Arabic countries like Qatar with 54% [24] and Iran with 50% [25]. According to some studies, the main reasons behind consanguineous marriages are, on the one hand, ensuring the safety and stability of women and their children, and on the other hand, preserving family physical properties [26,27]. The high rate of first cousins consanguineous unions, especially with the Father´s brother´s daughter, is similar to most other Arabic countries [28,29].

According to some studies, this preferential marriage with father´s brother´s daughter allows for maintaining family cohesion and safeguarding heritage [30-32]. Furthermore, the predominance of consanguineous unions from rural origin is consistent with what was reported by a similar study carried out in Israel, which demonstrated that consanguineous marriages are more frequent in the young generation that stayed in rural areas [33-35]. In addition, the fact that consanguinity was found to be significantly associated with low education level is confirmed by a study carried out on the Turkish population [36] but differs from what was reported by another study conducted in Pakistan according to which consanguineous unions increased in women with higher education level [37]. On the other hand, the significant association between consanguinity and isonymy in the present study could be explained by the fact that, in Islamic and Arabic countries, the transmission of the family name is possible only through men, and parents prefer to preserve their family name by marrying their daughters to a relative having the same name [38]. In concordance with the present work, many studies conducted in Morocco and elsewhere found that the isonymy-based consanguinity coefficient is higher than the genealogy-based one [39-43].

**Limitations:** the non-probabilistic nature of sampling makes the results not necessarily generalizable to this population. Furthermore, 38% of total questionnaires were eliminated from the study for unknown or undefined relationship between spouses, which reduced significantly the sample size.

## Conclusion

Through the present study, we showed that consanguinity remains a common practice in Tanger-Tetouan region (North Morocco) and that it is significantly associated with rural areas and low education level. Consequently, health authorities should make more efforts to raise the awareness of young people of the health disorders caused by consanguineous marriages. These preventive actions could help reducing health problems in descendants.

### What is known about this topic

- Consanguinity rates are known for many other Moroccan regions;
- Other Moroccan studies found out a predominance of first cousin consanguineous marriages, preferentially with the daughter of father's brother.

### What this study adds

- What makes investigating consanguinity interesting in this region of Morocco is the limited number of previous similar researches;
- This study confirms that consanguinity in North Morocco remains the highest compared with those reported in other Moroccan regions;
- This study contributes to raising the awareness of the population and the health authorities in Tanger-Tetouan region regarding the seriousness of the problem of consanguineous unions.
